# The semi-dwarfing gene *Rht-dp* from dwarf polish wheat (*Triticum polonicum* L.) is the "Green Revolution” gene *Rht-B1b*

**DOI:** 10.1186/s12864-021-07367-x

**Published:** 2021-01-19

**Authors:** Songyue Chai, Qin Yao, Xu Zhang, Xue Xiao, Xing Fan, Jian Zeng, Lina Sha, Houyang Kang, Haiqin Zhang, Jun Li, Yonghong Zhou, Yi Wang

**Affiliations:** 1 Triticeae Research Institute, Sichuan Agricultural University, Wenjiang, Chengdu, 611130 Sichuan China; 2 College of Resources, Sichuan Agricultural University, Wenjiang, Chengdu, 611130 Sichuan China; 3 Crop Research Institute, Sichuan Academy of Agricultural Sciences, Chengdu, 610066 Sichuan China

**Keywords:** Dwarf polish wheat, Homologous cloning, Molecular mapping, *Rht-B1b*, RNA-seq

## Abstract

**Background:**

The wheat dwarfing gene increases lodging resistance, the grain number per spike and harvest index. Dwarf Polish wheat (*Triticum polonicum* L., 2n = 4x = 28, AABB, DPW), initially collected from Tulufan, Xinjiang, China, carries a semi-dwarfing gene *Rht-dp* on chromosome 4BS. However, *Rht-dp* and its dwarfing mechanism are unknown.

**Results:**

Homologous cloning and mapping revealed that *Rht-dp* is the ‘Green Revolution’ gene *Rht-B1b*. A haplotype analysis in 59 tetraploid wheat accessions showed that *Rht-B1b* was only present in *T. polonicum*. Transcriptomic analysis of two pairs of near-isogenic lines (NILs) of DPW × Tall Polish wheat (*Triticum polonicum* L., 2n = 4x = 28, AABB, TPW) revealed 41 differentially expressed genes (DEGs) as potential dwarfism-related genes. Among them, 28 functionally annotated DEGs were classed into five sub-groups: hormone-related signalling transduction genes, transcription factor genes, cell wall structure-related genes, reactive oxygen-related genes, and nitrogen regulation-related genes.

**Conclusions:**

These results indicated that *Rht-dp* is *Rht-B1b*, which regulates pathways related to hormones, reactive oxygen species, and nitrogen assimilation to modify the cell wall structure, and then limits cell wall loosening and inhibits cell elongation, thereby causing dwarfism in DPW.

**Supplementary Information:**

The online version contains supplementary material available at 10.1186/s12864-021-07367-x.

## Background

Plant height is an important agronomic trait of crops. The discovery and utilization of semi-dwarfing genes in rice (*Oryza sativa*) and wheat (*Triticum aestivum*) triggered the “Green Revolution”, as dwarfism not only improves lodging resistance [1], but also increases the grain number per spike and harvest index [2, 3]. Increasing numbers of dwarf varieties of crops are being bred for production [4], and the dwarfing mechanisms in many crops are clearly revealed [5–7].

In wheat, 27 dwarfing genes including 32 alleles are present on chromosomes 2A, 2B, 2D, 3B, 4B, 4D, 5A, 5D, 6A, 7A, and 7B [8–16]. Twenty-two of those genes were discovered from hexaploid wheat, including *Rht1* (*Rht-B1b*), *Rht2* (*Rht-D1b*), *Rht8*, and *Rht12.* Those genes are widely utilized to breed new cultivars while only *Rht1* and *Rht2* have been cloned [6, 17, 18]. As the parent of hexaploid wheat, tetraploid wheat owns many dwarfing genes, for example, *Rht14*, *Rht15*, *Rht16*, *Rht18*, *Rht19,* and *Rht-R107* in *Triticum durum* [19, 20], *Rht22* in *T. turgidum* [21], contains *Rht-B1f* in *T. aethiopicum* [15], and *Rht-B1*^*IC12196*^ and *Rht-dp* in *T. polonicum* [10, 14]. Due to *T. polonicum* has a high 1000-grain weight and accumulates high concentrations of zinc and iron in grains, it is recommended to be a valuable material for wheat genetic improvement [22]. However, the details of its dwarfing genes, *Rht-dp* and *Rht-B1*^*IC12196*^, are still unknown.

As a gibberellin (GA)-insensitive semi-dwarfing gene, *Rht-dp* was identified from dwarf Polish wheat (DPW, *T. polonicum*) originally collected from Tulufan, Xinjiang province, China [10, 23]. Transcriptomic and proteomic analyses suggested that *Rht-dp* is probably involved in the phenylpropanoid pathway. It was found to reduce the contents of lignin, cellulose, and S-adenosyl-methionine, and increase the contents of flavonoids, which ultimately limits cell expansion and causes dwarfism [24]. Although those results indicated the potential mechanism of *Rht-dp*, the candidate gene of *Rht-dp* remained unknown. Genetic analysis of F_2_ population derived from the cross of DPW and tall Polish wheat (TPW) indicated that *Rht-dp* should be a recessive gene [10]. However, the separated threshold of plant height was significant larger than the plant height of DPW [10, 23], which implied that the effect of *Rht-dp* on reducing plant height might be partially covered by one or more non-allelic loci. Further study mapped *Rht-dp* onto chromosome 4BS between the SSR markers *Xgpw3017* and *Xwmc511*, and suggested that *Rht-dp* may be an alternative allele at the *Rht-B1* locus [10]. However, due to the limited numbers of F_2_ plants and molecular markers used in the analysis, a genomic alignment against the genome of *Triticum aestivum* ‘Chinese Spring’ (IWGSC RefSeq v1.0) (International Wheat Genome Sequencing Consortium, 2018) indicated that the region between *Xgpw3017* and *Xwmc511* did not include the *Rht-B1* locus. Additionally, *Rht-B1b* and its alleles are semi-dominant genes [6, 25, 26]. Thus, we can’t confirm whether *Rht-dp* is *Rht-B1b* or its allele, or a new gene.

*Rht-B1b* encodes a premature DELLA protein, which prevents GID1 from binding to its target [12]. The premature DELLA protein truncates the GA response, resulting in dwarfism. *Rht-B1b* originates from the native Japanese dwarf variety ‘Norin 10’ [27]. However, it was successfully transferred from ‘Norin 10’ to ‘Cando’ in the 1960s and widely used in durum wheat breeding [28]. Meanwhile, three alleles of *Rht-B1b*, *Rht-B1f*, *Rht-R107,* and *Rht19*, were also discovered from *T. aethiopicum* and *T. durum*, respectively [15, 19]. Although DPW is originally collected from Tulufan, Xingjiang, China [23] and the progenitor of *T. polonicum* is neither ‘Norin 10’, *T. aethiopicum* nor *T. durum* [10, 29], we still hypothesized that the candidate gene of *Rht-dp* may be *Rht-B1b* or its one of alleles, because only *Rht-B1b* and its alleles as dwarfing genes have been found on 4BS to date [10, 23, 28, 30].

To test this hypothesis and to understand the dwarfing mechanism of *Rht-dp* in DPW, we firstly cloned *Rht-B1* to investigate sequence differences in *Rht-B1* between DPW and TPW. Secondly, we developed and applied a specific molecular marker of *Rht-B1* and SSR markers on 4BS to genetically confirm the candidate region using three recombinant inbred lines (RILs). Thirdly, two pairs of near-isogenic line (NIL) obtained from the F_7_ population of DPW × TPW were conducted transcript analyses to reveal the molecular mechanism of *Rht-dp*; meanwhile, F_1_ plants and a F_2_ population derived from the cross of a pair of NIL were developed for further genetic analysis. Finally, we conducted a haplotype analysis of *Rht-dp* to reveal the natural distribution among 59 tetraploid wheat accessions.

## Methods

### Plant materials and growth conditions

The DPW and TPW lines were originally collected from Tulufan, Xinjiang province, China, by Prof. Chi Yen and Junliang Yang (Sichuan Agricultural University, China) in the 1980s. The F_1_ population of DPW × TPW and the F_2_ population (401 plants) derived from DPW × TPW were individually developed for trait investigation. Two RIL populations (F_7_ including 330 lines and F_8_ including 300 lines) derived from DPW × TPW, and a RIL population (F_6_ including 194 lines) derived from DPW × Jianyangailanmai (AABB, 2n = 4x = 28, *T. turgidum* L., Ailanmai), were developed for gene mapping. Two pairs of NILs (D_60/T_58, and D_33/T_35, D and T represent dwarf and tall phenotype, respectively) derived from two heterozygous F_7_ lines were selected for transcript analyses. Meanwhile, F_1_ plants and a F_2_ population (244 plants) derived from the cross of D_60 and T_58 were developed for trait investigation. The haplotype analysis was conducted using 59 tetraploid wheat accessions (Table S1).

DPW, TPW and their F_1_ plants and F_2_ population were grown at the Wenjiang experimental field of Sichuan Agricultural University, Chengdu, China, in the 2011–2012 (from October 2011 to June 2012) and 2012–2013 (from October 2012 to June 2013) wheat growing seasons. The F_7_ and F_8_ RIL populations of DPW × TPW were grown at two experimental fields (Wenjiang and Chongzhou) of Sichuan Agricultural University (Chengdu, China) in the 2017–2018 (from October 2017 to June 2018) and 2018–2019 (from October 2018 to June 2019) wheat growing seasons, respectively. The F_6_ RIL population, the F_1_ plants of D_60 × T_58, two pairs of NILs, and 59 tetraploid wheat accessions were grown at the Wenjiang experimental field in the 2018–2019 (from October 2018 to June 2019) wheat growing season. The F_2_ population of D_60 × T_58 was grown at the Wenjiang experimental field in the 2019–2020 (from October 2019 to June 2020) wheat growing season. Each line was planted with 20 plants per row. The rows were 2 m long and the spacing between rows was 30 cm.

### Phenotypic measurements and analysis

Plant height, spike length, and stem length were measured at maturity. We selected three individual plants per line and calculated the average value. Data was analysed using SPSS software (version 18.0; SPSS, Chicago, IL, USA) Figures were drawn using SigmaPlot software (version 12.0; Systat, Point Richmond, CA, USA).

### Homologous cloning of *Rht-B1*

According to the genomic sequence of *T. aestivum* cv. ‘Chinese Spring’ (IWGSC RefSeq v1.0), a pair of *Rht-B1*-specific primers (forward: 5*′*-CGATGCCGTC TACAACTACT-3*′*; reverse: 5*′*-CAACTCCTAGATCGGGAAACTT-3*′*) was designed using Beacon designer software (version 7.0; Premier Biosoft International, Palo Alto, CA, USA). These primers were used to amplify the full-length *Rht-B1* sequence from DPW and TPW. Each PCR reaction mixture contained 2 μl DNA, 2 μl mixture of forward and reverse primers (4 pmol/μl), 2 μl dNTP (2.5 mM/μl), 1 μl Ex-Taq polymerase (5 U/μl), 2 μl MgCl_2_ (2.5 mM/μl), 2.5 μl 10× PCR buffer, and 13.5 μl ddH_2_O. The PCR amplification conditions were 95 °C for 5 min, 40 cycles (95 °C for 30 s, 58 °C for 30 s, and 72 °C for 2 min), and final extension at 72 °C for 10 min. Each amplified fragment was cloned into the pMD19-T vector for sequencing. Differences in *Rht-B1* sequences between DPW and TPW were detected in an alignment analysis using Vector NTI software (version 11.5.1; Invitrogen, Carlsbad, CA, USA).

### Exploitation of indel marker of *Rht-B1* for mapping

According to the sequence differences in *Rht-B1* between DPW and TPW, a pair of *Rht-B1*-specific primers (*Rht-B1 Indel-*F: 5*′*-GGCGGGAGATCGAAGTAC-3′, *Rht-B1 Indel-*R: 5′-GACACCGTGCACTACAAC-3′) was designed using Beacon designer software.

### Exploitation of SSR markers on 4BS for mapping

According to the genomic sequence of 4BS of *T. aestivum* cv. ‘Chinese Spring’ (IWGSC RefSeq v1.0) (http://plants.ensembl.org/), microsatellites were predicted using the MIcroSAtellite identification tool (https://webblast.ipk-gatersleben.de/misa/) [31, 32]. Beacon designer software was used to design SSR markers (Table S2).

### Genotyping and genetic mapping

Genomic DNA was extracted from DPW, TPW, Ailanmai and the mapping populations RIL_6_ (DPW × Ailanmai), RIL_7_ and RIL_8_ (DPW × TPW) using a plant genomic DNA kit (TIANGEN BIOTECH, Beijing, China). Each PCR reaction mixture contained 1 μl DNA, 2 μl mixture of forward and reverse primers (4 pmol/μl), 1.5 μl dNTP (2.5 mM/μl), 0.5 μl Taq polymerase (5 U/μl), 1.5 μl MgCl_2_ (2.5 mM/μl), 2 μl 10× PCR buffer, and 11.5 μl ddH_2_O. The PCR amplification conditions were 95 °C for 5 min, 35 cycles (95 °C for 45 s, 58 °C for 45 s, and 72 °C for 45 s), and final extension at 72 °C for 7 min. The PCR products were separated on 8% polyacrylamide gels. The polymorphic bands between the parents were used to genotype individual lines of the mapping populations.

The *Rht-B1 Indel* marker and 15 polymorphic SSR markers were first used for genetic mapping of *Rht-dp* in the F_7_ RIL population*.* Then, *Rht-B1Indel* and its four flanking SSR markers (*Xgpw2994.1*, *Xgpw3128.1*, *Xgpw3427.1*, and *Xgpw4800.1*) were further used to confirm the candidate region in the F_8_ RIL and F_6_ RIL populations. The F_7_ RIL population was hybridized on the wheat 55 K SNP array by CapitalBio Technology (Beijing, China) (unpublished data).

Linkage analysis was performed using the JoinMap software (version 4.0; Kyazma BV, Wageningen, Netherlands) with a logarithm of odds (LOD) threshold of 3.0. The Kosambi mapping function was used to convert the recombination frequencies into genetic distances (cM) [33].

### Haplotype analysis of Rht-B1 in 59 tetraploid wheat accessions

Genomic DNA was extracted from each tetraploid wheat accession using a plant genomic DNA kit (TIANGEN BIOTECH, Beijing, China), and PCR amplification was performed as described in the section “Homologous cloning of *Rht-B1*”. The amino acid sequence was deduced using ExPASy software (http://web.expasy.org/ translate/). All sequences were aligned using Vector NTI software (Invitrogen). A phylogenetic tree was constructed using the neighbour-joining algorithm in MEGA5 (https://www.megasoftware.net/).

### Expression analysis of *Rht-B1b*

Tissues at the three growth stages (jointing, booting, and grain filling stages) were collected, including roots, basal stems, leaf sheaths, leaf blades, young leaves, lower leaf blades, first and second internodes, flag leafs, and spikes. The collected tissues were snap-frozen in liquid nitrogen and stored at − 80 °C until RNA extraction. Total RNA was extracted using a Plant RNA Kit (Omega Bio-Tek, American). cDNA was synthesized using the M-MLV First Strand cDNA Synthesis kit (Invitrogen).

Quantitative real-time PCR (qPCR) was performed on the CFX-96 system as described by Wang et al. using a pair of *Rht-B1b*-specific primers (forward: 5′-GGCGGGAGATCGAAGTAC-3′; reverse: 5′-GACACCGTGCACTACAAC-3′) [34]. To normalize gene expression levels, the *Actin* gene was used as the reference gene [34]. Relative expression levels were calculated according to the 2^ΔΔCt^ method using the CFX Manager (version 3.1; Bio-Rad, Hercules, CA, USA).

### Transcript analysis of two pairs of NILs

#### Sample collection

At the booting stage, the first internode was collected individually from two pairs of NILs, and then snap-frozen in liquid nitrogen and stored at − 80 °C until RNA extraction.

#### RNA extraction, library preparation and sequencing

Total RNA was isolated as described above, and RNA degradation and contamination were monitored on 1% agarose gels. A NanoPhotometer® spectrophotometer (Implen GmbH, Munich, Germany) RNA purity was used to check RNA purity. The mRNA was purified from total RNA using poly-T oligo-attached magnetic beads and divided into short fragments using NEBNext First Strand Synthesis Reaction Buffer (5×) (New England Biolabs, Ipswich, MA, USA). The cDNA was synthesized using the fragments as templates and then purified and resolved with EB buffer for the end-repair step and addition of a single adenine (A) nucleotide. To select cDNA fragments 250 ~ 300 bp in length, the library fragments were purified with the AMPure XP system (Beckman Coulter, Beverly, CA, USA), and suitable fragments were chosen for a PCR amplification. The PCR products were purified (AMPure XP system) and the library quality was assessed using the Agilent Bioanalyzer 2100 system. The prepared libraries were sequenced on the Illumina Hiseq platform.

#### RNA-seq data analysis

Raw data (raw reads) of in fastq format were first processed using in-house perl scripts. In this step, clean data (clean reads) were obtained by removing reads containing adapters, reads containing poly-N, and low-quality reads from the raw data. All the downstream analyses were conducted using clean, high-quality data.

The Chinese Spring (IWGSC RefSeq v1.0) reference genome and gene model annotation files were downloaded from the genome website (https://urgi.versailles. inra.fr/download/iwgsc/IWGSC_RefSeq_Assemblies/v1.0). The D genome sequences were excluded from the reference before mapping the processed reads of the tetraploid lines (A and B genomes). An index of the Chinese Spring reference genome was built using Bowtie v2.2.3 and paired-end clean reads were aligned to the reference genome using TopHat v2.0.12. HTSeq v0.6.1 was used to count the number of reads mapped to each gene. The mean fragments per kilobase of transcript per million mapped reads (FPKM) value for each gene was calculated based on the length of the gene and the number of reads mapped to it [35].

#### Differential expression analysis

Read counts were adjusted by the edgeR program package through one scaling normalized factor. Analysis of differential gene expression between two pairs of NILs (D33/T35 and D60/T58) was performed using the DEGSeq R package. The *P* values were adjusted using the Benjamini and Hochberg method. A corrected *P*-value of 0.005 and log2 (fold change) of 1 were set as the thresholds for significantly different gene expression.

#### QPCR for validation

Two differentially expressed genes Auxin-repressed protein (ARP) and *L-ascorbate oxidase homolog* (*ASCO*) from RNA-Seq were verified by qPCR, and their gene-specific primers sequences were *APR* (forward: 5′-ATTAAGCAGTCGCCG TCGAT-3′; reverse: 5′-TCGCTGTAAAGCCAG TCGTA − 3′) and *ASCO* (forward: 5′-AATGGCAATAGGTTCACAGTAGA-3′; reverse: 5′-CTTCACGAGGAACGAGT AGG-3′), respectively.

## Results

### Phenotype of plants harbouring *Rht-dp*

**Fig. 1:**
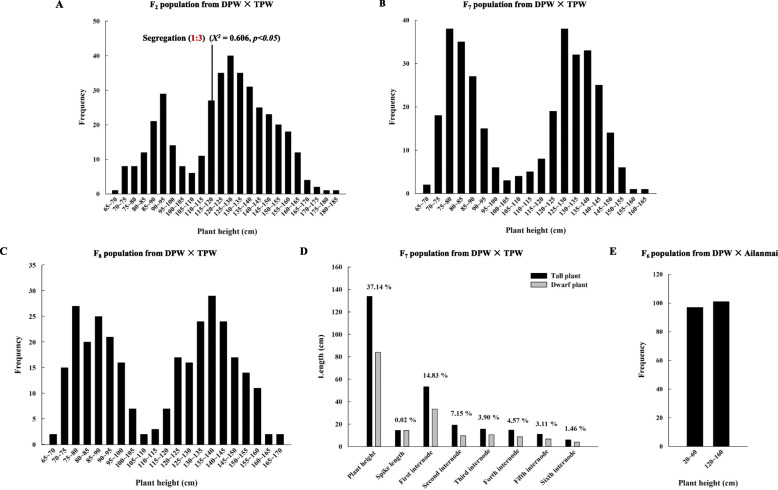
Phenotypic characterization. **a** frequency distribution of plant heights in the F_2_ population from DPW × TPW; **b** frequency distribution of plant heights in the DPW × TPW F_7_ population; **c** frequency distribution of plant heights in the DPW × Ailanmai F_6_ population; **d** plant height, the lengths of spike and each internode of DPW × TPW NILs F_7_ at the maturate stage; **e** frequency distribution of plant heights in the DPW × TPW F_8_ population

The average heights of DPW and TPW were 91.52 ± 2.97 cm and 189.88 ± 1.72 cm, respectively. No significant difference in plant heights between F_1_ plants (179.12 ± 3.65 cm) and TPW was observed (Fig. S1). The plant heights of F_2_ plants ranged from 65 to 185 cm. According to the frequency distribution of plant height, F_2_ plants were separated into two groups of dwarf and tall phenotypes at 110 cm (Fig. 1a). The dwarf and tall phenotype groups included 107 and 294 plants, respectively, consistent with the expected Mendelian segregation ratio of 1:3 (*X*^*2*^ = 0.606, *p* < 0.05). These results validate that *Rht-dp* should be a major recessive gene. However, the separated threshold of plant height with 110 cm was significantly larger than the plant height of DPW with 91.52 ± 2.97 cm, which implied that the effect of *Rht-dp* on reducing plant height might be partially covered by one or more non-allelic loci.

To fine-map *Rht-dp*, two RIL populations including 330 F_7_ and 300 F_8_ plants were constructed. The plant heights of F_7_ and F_8_ plants ranged from 65 to 165 cm (Fig. 1b) and from 65 to 170 cm (Fig. 1c), respectively. For the F_7_ population, the average heights of dwarf and tall phenotypes were 84.07 ± 1.97 cm and 133.75 ± 2.01 cm, respectively. Compared with the tall phenotype, the lines harbouring *Rht-dp* showed a reduction in plant height of up to 37.14%. The reduced plant height was because of the shortened first internode (by 14.83%), second internode (by 7.15%), and basal internode (by 1.46%), but the length of the spike was not affected (Fig. 1d). These results indicate that *Rht-dp* reduces plant height mainly by restricting elongation of the first and second internodes at the booting stage.

To validate the candidate region of *Rht-dp* in a different genetic background, an F_6_ RIL population including 194 lines derived from DPW × Ailanmai was constructed. The average height of Ailanmai was 100.98 ± 0.37 cm. Ailanmai has a recessive dwarfing gene *Rht22*, which has an additive effect with *Rht-dp*. The RIL population was grouped into dwarf and tall phenotypes with heights ranging from 20 to 60 cm and from 120 to 160 cm, respectively (Fig. 1e).

### Characterization of *Rht-dp* in F_1_ plants and F_2_ population derived from the cross of a pair of NIL

**Fig. 2:**
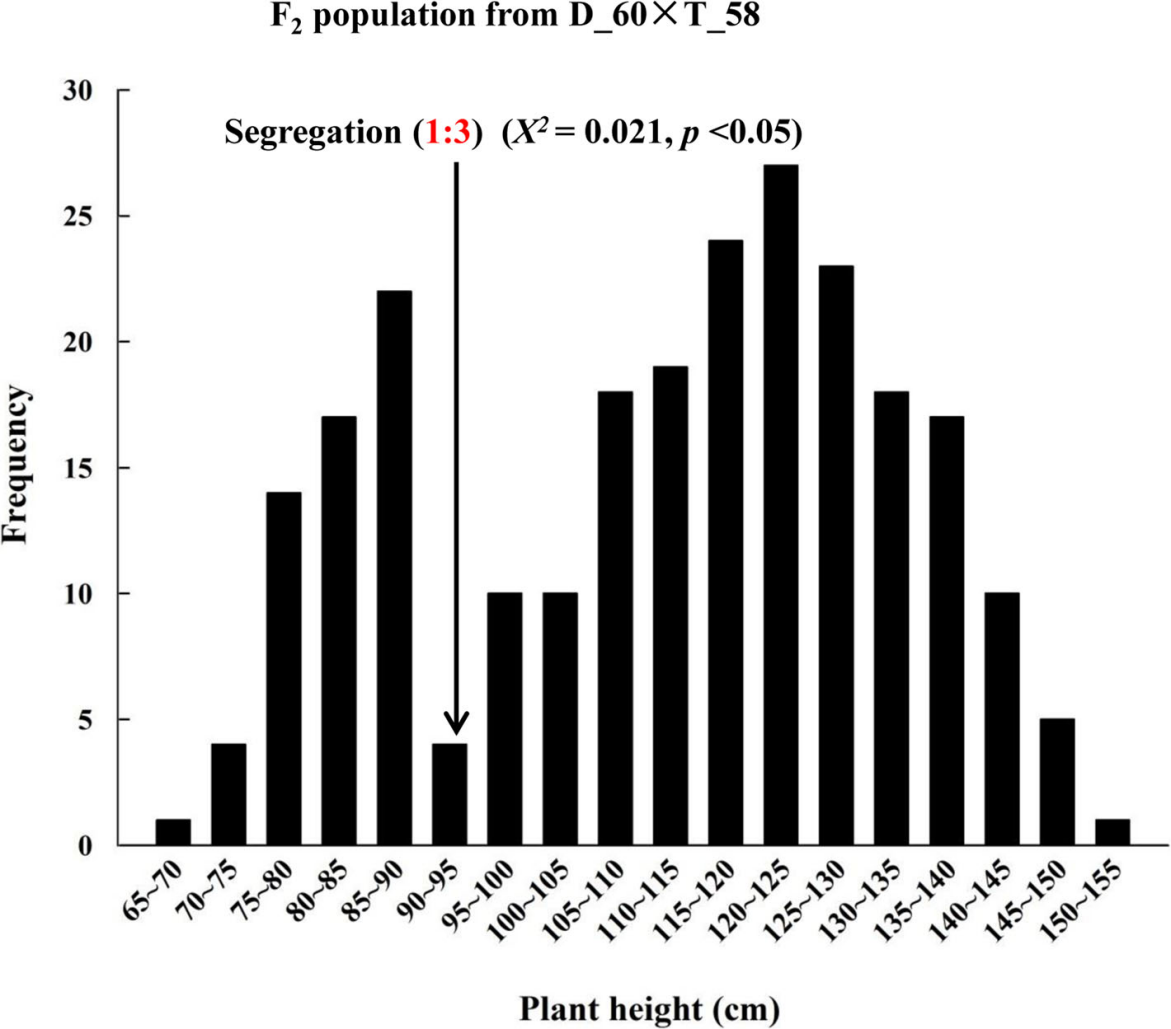
Frequency distribution of plant heights in the F_2_ population from D_60 × T_58

Since genetic analysis suggested that the effect of *Rht-dp* on reducing plant height was probably influenced by one or more non-allelic loci derived from TPW, a QTL analysis was performed on the F_7_ RIL population using the wheat 55 K SNP array. Beside of a major-locus on 4BS (*Rht-dp*) derived from DPW caused dwarfism, a micro-locus on 5A derived from TPW heightened plant was detected (unpublished data). To further confirm the information of *Rht-dp*, we measured the plant height of F_1_ plants and F_2_ population derived from the cross of a pair of NIL (D_60 and T_58). The average heights of D_60 and T_58 were 93.52 ± 1.83 cm and 159.67 ± 2.72 cm, respectively; the average plant height of F_1_ was 123.23 ± 2.55 cm. Compared with T_58, F_1_ plants harbouring *Rht-dp* showed a reduction in plant height up to 22.82%. The plant heights of F_2_ plants ranged from 65 to 155 cm. According to the frequency distribution of plant height, F_2_ plants were separated into two groups of dwarf and tall phenotypes at 95 cm (Fig. 2). The dwarf and tall phenotype groups included 62 and 182 lines, respectively, consistent with the expected Mendelian segregation ratio of 1:3 (*X*^*2*^ = 0.021, *p* < 0.05). Meanwhile, the separated threshold of plant height with 95 cm was similar to the plant height of D_60 with 93.52 ± 1.83 cm. These results indicate that the dwarfing gene of *Rht-dp* should be a single semi-dominant gene, and further imply that the candidate gene is *Rht-B1b*.

### Differences in sequence of *Rht-B1* between DPW and TPW

**Fig. 3:**
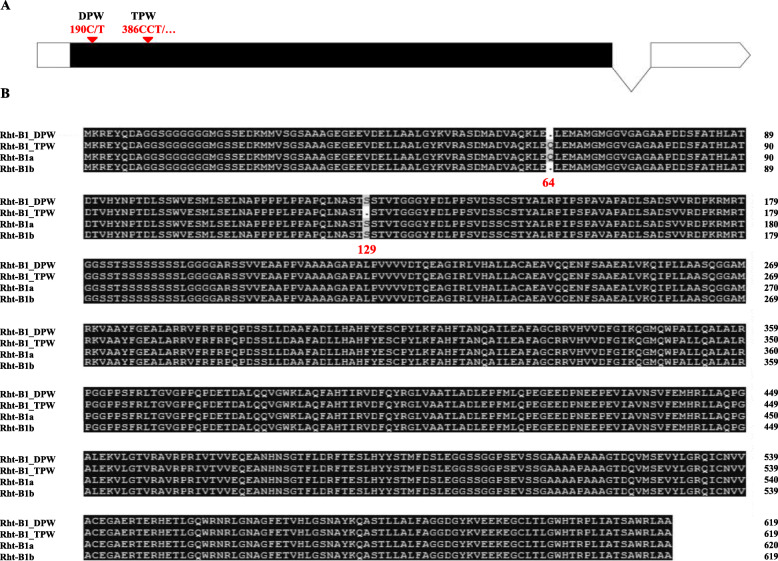
Sequences of Rht-B1 in DPW and TPW. **a**: nucleotide mutations of *Rht-B1* in DPW and TPW; **b**: amino acid mutations of Rht-B1 in DPW and TPW

To test the implication that the candidate gene of *Rht-dp* is *Rht-B1b* or one of its alleles, the sequences of *Rht-B1* were cloned from DPW and TPW. Sequence analysis showed that *Rht-B1* of DPW is *Rht-B1b*, with a single nucleotide change from C to T at the nucleotide position 190 when compared with *Rht-B1a* (Fig. 3a) that results in a premature termination codon at amino acid position 64 (Fig. 3b). Although *Rht-B1* of TPW did not have this single nucleotide change from C to T at nucleotide position 190, it had a three-nucleotide deletion at nucleotide position 386–388 when compared with *Rht-B1a* (Fig. 3a), resulting in a serine (S) deletion at amino acid position 129 (Fig. 3b). These results imply that the candidate gene of *Rht-dp* might be *Rht-B1b*. An *Rht-B1 Indel* marker was developed from the three-nucleotide deletion of *Rht-B1* in TPW for further analysis.

### Mapping of *Rht-dp*

**Fig. 4:**
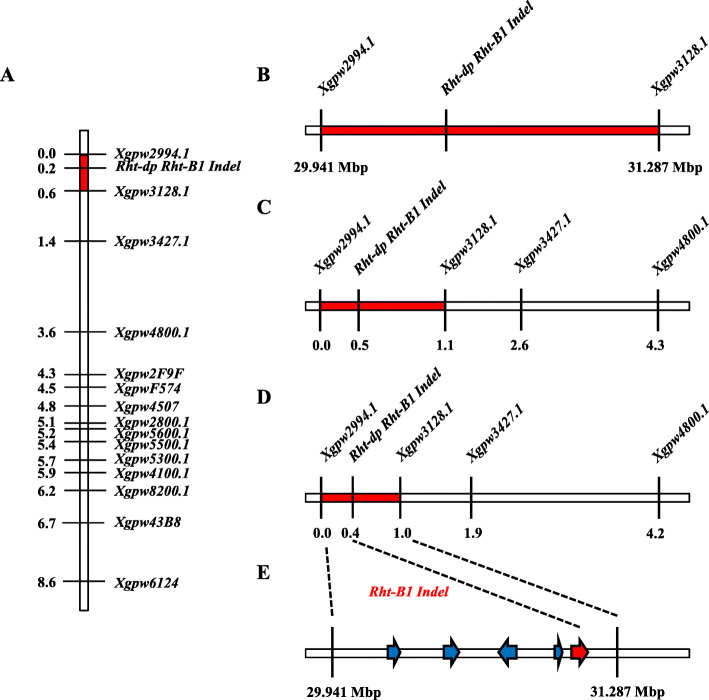
Mapping of *Rht-dp*. **a**: mapping of *Rht-dp* in the DPW × TPW RILs F_7_; **b**: mapping of *Rht-dp* in the F_2_ population from D_60 × T_58; **c**: mapping of *Rht-dp* in DPW × Ailanmai RILs F_6_; **d**: mapping of *Rht-dp* in DPW × TPW RILs F_8_; **e**: candidate genes between SSR markers *Xgpw2994.1* and *Xgpw3128.1*

To confirm that the candidate gene of *Rht-dp* is *Rht-B1b*, the *Rht-B1Indel* marker was first used to determine whether *Rht-B1* was tightly linked with *Rht-dp*. Genetic mapping analyses confirmed that the *Rht-B1Indel* marker completely co-segregated with *Rht-dp* in three RIL populations and a F_2_ population derived from a pair of NIL (Fig. 4).

To further confirm that *Rht-B1b* is located in the candidate region of *Rht-dp*, 190 pairs of SSR markers were exploited according to the genome reference of 4BS (Table S2). Fifteen pairs of SSR markers exhibited polymorphism between DPW and TPW, and were linked with *Rht-dp* in the F_7_ RIL population. Of them, two SSR markers, *Xgpw2994.1* and *Xgpw3128.1*, were tightly linked with *Rht-dp* with a genetic distance of 0.6 cM (Fig. 4a; Table S3). *Xgpw2994.1* and *Xgpw3128.1* were further confirmed as tightly linked markers flanking *Rht-dp* in the F_2_ population derived from NIL (Fig. 4b), and the F_6_ (Fig. 4c) and F_8_ (Fig. 4d) RIL populations (Table S3).

Based on the gene annotation of wheat 4BS from 29.94 to 31.29 Mbp, flanked by *Xgpw2994.1* and *Xgpw3128.1*, there were five potential genes: *TraesCS4B01G042700* (encodes a teosinte branched 1 protein), *TraesCS4B01G042800* (encodes an uncharacterized protein), *TraesCS4B01G042900* (a RING finger protein), *TraesCS4B01G043000* (EamA-like transporter family)*,* and *TraesCS4B01G043100* (*Rht-B1* encodes a DELLA protein) (Fig. 4e). Apart from *Rht-B1*, sequence difference of other four genes (primers listed in Table S4) between DPW and TPW was not found. These results indicate that the candidate gene of *Rht-dp* should be *Rht-B1b*.

### Expression patterns of *Rht-B1b* in DPW

**Fig. 5:**
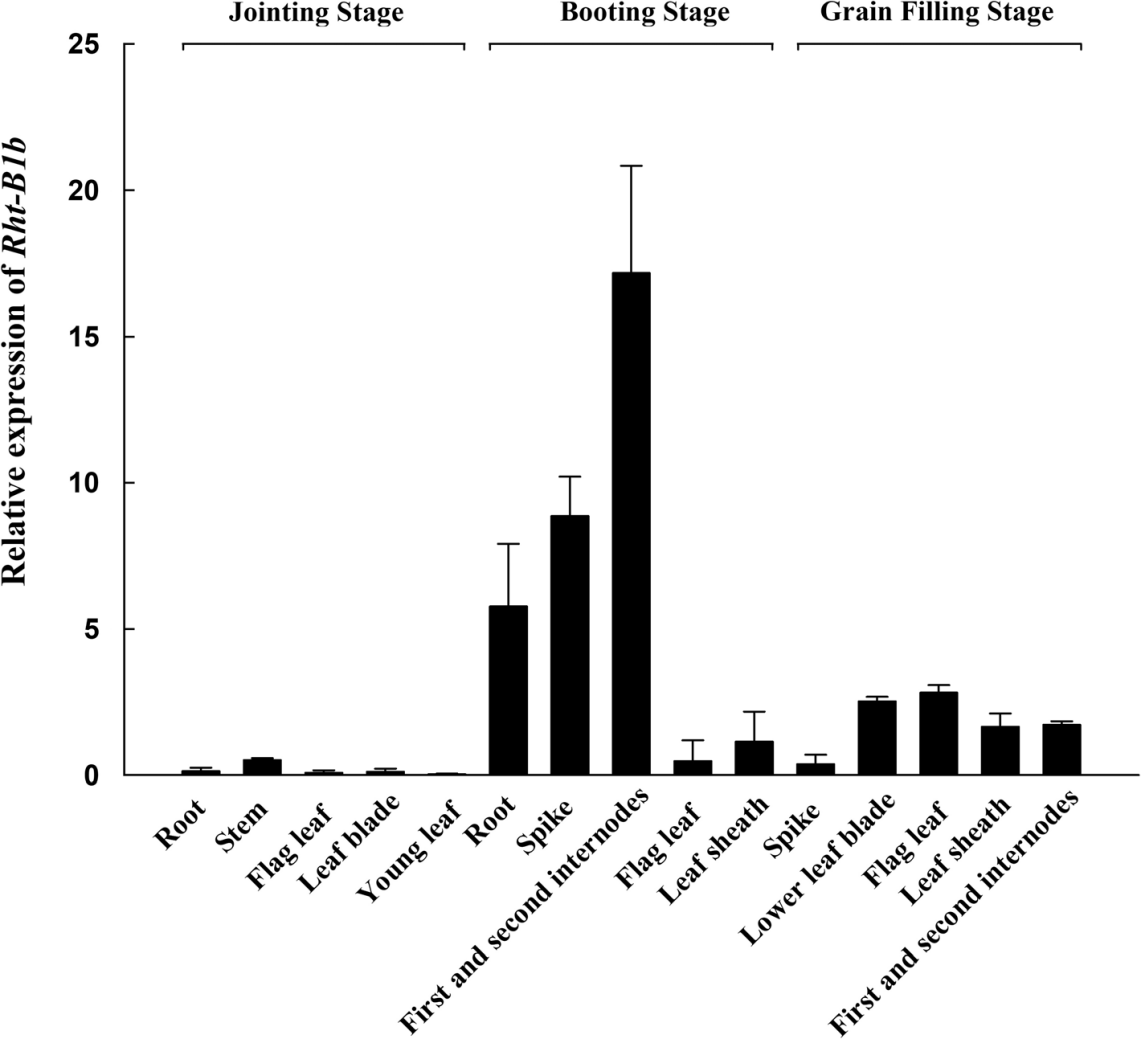
Expression patterns of *Rht-dp* in various wheat tissues at jointing, booting, and grain filling stages

To confirm that *Rht-B1b* reduces plant height via its effects on elongation of the first and second internodes at the booting stage, the transcriptional patterns of *Rht-B1b* were investigated in different DPW tissues at the jointing, booting, and grain-filling stages. *Rht-B1b* was mainly expressed in the first and second internodes at the booting stage, and at dramatically higher levels in those tissues than in other tissues at the jointing, booting, and grain-filling stages (Fig. 5).

### Allelic variations of Rht-B1 in tetraploid wheat accessions

**Fig. 6:**
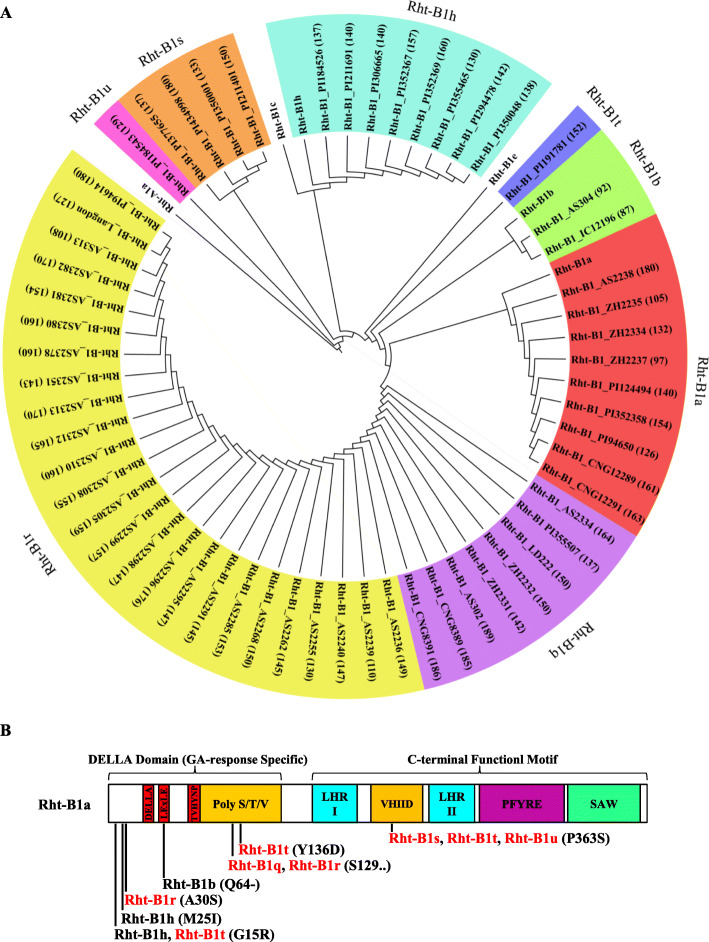
Haplotype analysis of Rht-B1 in 59 tetraploid wheat accessions. **a** phylogenetic relationship of *Rht-B1* alleles; **b** nonsynonymous mutations in Rht-B1

*Rht-B1b* has never been found in spontaneous tetrapolid accession. Since *Rht-B1b* is the candidate gene of *Rht-dp* in DPW, the haplotypes of *Rht-B1b* in 59 tetraploid wheat accessions were analysed. Among them, five accessions were dwarf phenotypes including two *T. turgidum* (AS313 and AS2239), two *T. polonicum* [AS304 (DPW) and IC12196], and one *T. durum* (ZH2237). The 59 sequences cloned from the 59 tetraploid wheat accessions were grouped into eight types. *Rht-B1b* was only obtained from two *T. polonicum* (DPW and IC12196) accessions; and *Rht-B1t* and *Rht-B1u* were only obtained from *T. turgidum. Subsp. dicoccon* (PI191781) and *T. turgidum. Subsp. Turanicum* (PI184543), respectively. Of them, five novel types (named *Rht-B1q–B1u*, respectively) were identified by comparison with *Rht-B1a* (Fig. 6b). Rht-B1q contained an S deletion at position 129 (S129); Rht-B1r carried a mutation at position 30 (A30S) and an S deletion at position 129 (S129); Rht-B1s contained a mutation at position 363 (P363S). Rht-B1t had two mutations at positions 15 (G15R) and 363 (P363S). Rht-B1u also had two mutations at positions 136 (Y136D) and 363 (P363S) (Fig. 6b).

Among these variations, Rht-B1q had the highest frequency (43.9%). The frequencies of Rht-B1a, Rht-B1b, Rht-B1h, Rht-B1r, Rht-B1s, Rht-B1t, and Rht-B1u were 15.3, 3.4, 13.6, 13.6, 6.8, 1.7, and 1.7%, respectively.

### Dwarfism-related DEGs induced by DELLA mutant Rht-B1b

To understand the molecular networks of *Rht-B1b*, the DEGs induced by the DELLA mutation *Rht-B1b* in the first internode of two pairs of NILs were investigated. A total of 41 DEGs was obtained, 30 of which were successfully functionally annotated (Table S5). Twenty-eight DEGs were further classed into five sub-groups; hormone-related signalling transduction genes, transcription factor genes, cell wall structure-related genes, reactive oxygen-related genes, and nitrogen regulation-related genes (Table 1). Among the hormone-related signal transduction genes, two brassinolide (BR) signal-related genes *serine carboxypeptidase II-3* (*SCP*) and *cytochrome P450 710A1* (*CYP450*) were down-regulated; and genes encoding salicylic acid (SA)-binding protein 2 and ARP were up-regulated in the dwarf phenotype. The only down-regulated transcription factor gene was *MybAS2*. Fifteen DEGs were grouped into cell wall structure-related genes (seven pectin-related genes and eight xylan acetylation-related genes). In the dwarf phenotype, five pectin-related genes [encoding a pectate lyase 15 (PEL15), three subtilisin-like protease (SBT1.7), and an alpha-galactosidase (α-Gal)] involved in pectin modification were down-regulated; while all eight xylan acetylation-related genes, including three *GDSL esterase/lipase* genes, two *ESKIMO* genes, *IRX15-L*, *ALTERED XYLOGLUCAN 4-like* (*AXY-L*), and an uncharacterized acetyltransferase gene were up-regulated. For the reactive oxygen-related genes, *plant cysteine oxidase 2* (*PCO2*) and *ASCO* were down-regulated; and genes encoding germin-like protein 5–1 (GLP) and blue copper protein (BCP) were up-regulated in the dwarf phenotype. For nitrogen assimilation-related genes, two *phosphoenolpyruvate carboxylase kinase 2* (*PPCK2*) genes and *early nodulin* (*ENOD*) were down-regulated; and *asparagine synthetase* (*APS*) was up-regulated in the dwarf phenotype. We verified the expression of *ARP* and *ASCO* in the first and second internodes at the booting stage (Fig. S2).

**Table 1:** Dwarfism-related DEGs induced by DELLA mutant *Rht-dp*

Gene ID	Description	Fold change of transcript	
		D_60/T_58	D_33/T_35
Hormone-related signaling transduction genes			
*TraesCS2B01G157100*	Serine carboxypeptidase II-3	−32	−20
*TraesCS3B01G167400*	Cytochrome P450 710A1	−25	−18
*TraesCS2B01G471800*	Salicylic acid-binding protein 2	28	39
*TraesCS4B01G070300*	Auxin-repressed 125 kDa protein	12	15
Transcription factor			
*TraesCS1B01G055200*	Myb-related protein MYBAS2	−13	−26
Cell wall structure-related genes			
*Pectin-related genes*			
*TraesCS2A01G016500*	Pectate lyase 15	−17	−29
*TraesCS4A01G237500*	Subtilisin-like protease SBT17	−20	−22
*TraesCS4B01G077600*	Subtilisin-like protease SBT17	−29	−17
*TraesCS6A01G339400*	Subtilisin-like protease SBT17	−14	−12
*TraesCS6B01G332900*	Alpha-galactosidase	−16	−11
*TraesCS1B01G249000*	(1–3,1–4)-beta-D-glucanase	21	28
*TraesCS2A01G341400*	Sugar transport protein 5	11	14
*Xylan acetylation-related genes*			
*TraesCS3A01G258100*	GDSL esterase/lipase	15	11
*TraesCS3B01G290800*	GDSL esterase/lipase	13	10
*TraesCS7B01G250700*	GDSL esterase/lipase	13	23
*TraesCS4A01G110000*	ESKIMO 1	14	10
*TraesCS4B01G194100*	ESKIMO 1	17	10
*TraesCS6A01G131900*	IRX15-like	13	11
*TraesCS7A01G191700*	ALTERED XYLOGLUCAN 4-like	17	11
*TraesCSU01G204900*	Uncharacterized acetyltransferase	28	16
Reactive oxygen-related genes			
*TraesCS5A01G025200*	Plant cysteine oxidase 2	−11	−15
*TraesCS7A01G459400*	L-ascorbate oxidase homolog	−34	−51
*TraesCS3A01G165500*	Germin-like protein 5–1	16	22
*TraesCS6A01G315800*	Blue copper protein	13	12
Nitrogen regulation-related genes			
*TraesCS6A01G375800*	Phosphoenolpyruvate carboxylase kinase 2	−12	−20
*TraesCS6B01G413500*	Phosphoenolpyruvate carboxylase kinase 2	−12	−15
*TraesCS7A01G091800*	Early nodulin-93	−40	−12
*TraesCS3B01G385400*	Asparagine synthetase	11	12

## Discussion

The GA-insensitive dwarfing gene *Rht-B1b* is the predominant source of the semi-dwarf growth habit of wheat plants grown in parts of Northern Europe [36], the Mid and Lower Yangtze Valley Autumn-sown Spring Wheat Region in China [37], and the Great Plains Hard Winter Wheat Region in the USA [38]. Because *Rht-B1b* significantly decreases plant height to reduce plant lodging and increase wheat yield [37, 39], it has been introduced into tetraploid wheat *T. durum* for dwarf breeding [28]. However, it is well known that the progenitor of *T. polonicum* is not Norin 10 or *T. durum*. Additionally, DPW was originally collected from Tulufan, Xingjiang, China [23]. Thus, the dwarfing gene *Rht-dp* of *T. polonicum* cannot be derived from Norin 10 or *T. durum*. However, our results show that the candidate gene *Rht-dp* of DPW is *Rht-B1b*. This conclusion is supported by the following evidences: (1) *Rht-dp* is a single semi-dominant dwarfing gene, as is *Rht-B1b* [6]. (2) *Rht-dp* and *Rht-B1b* reduce plant height mainly via reducing the length of the first and second internodes (Fig. 1d), and their effects on reducing plant height are similar with 22% [18, 39]. (3) The sequence of *Rht-B1* of DPW is the same as that of *Rht-B1b* (Fig. 3). (4) Mapping work revealed that the candidate region of *Rht-dp* was between SSR markers *Xgpw2994.1* and *Xgpw3128.1* (Fig. 4b-d). This region contains five potential genes including *Rht-B1* (Fig. 4e); except of *Rht-B1*, other four genes have no sequence difference between DPW and TPW. (5) The *Rht-B1 Indel* marker developed based on the sequence difference of *Rht-B1* between DPW and TPW is completely co-segregated with *Rht-dp* in a F_2_ population derived from NIL and three RIL populations (Fig. 4). In the haplotype analysis, *Rht-B1b* was only obtained from *T. polonicum* (Fig. 6a), implying that it might originate from this species, or might be introduced into *T. polonicum* from other unknown species but not *T. aethiopicum* and *T. durum*.

In wheat, *Rht-B1b* encodes a DELLA mutant protein resembling the SLRL1 protein. Its accumulation represses GA-regulated growth and developmental responses and causes the typical semi-dwarf phenotype [6, 40]. DELLA not only regulates the expression of downstream genes but also interacts with DNA-binding transcription factors. Our transcript analysis identified 28 DEGs regulated by the DELLA mutant *Rht-B1b* involved in the processes of nitrogen assimilation, oxidation-reduction, modification of the cell wall components and structures, and hormone-related signal transduction (Table 1). However, this list of DEGs only slightly overlaps with those identified in previous studies, suggesting that the effects of DELLA on transcription depend on the species, organ, and developmental context [41–44]. Since *Rht-B1b* is mainly expressed in the first and second internodes (Fig. 5) to dramatically reduce their lengths at the booting stage in DPW (Fig. 1d), we explored the molecular network of *Rht-dp* by conducting a transcript analysis of the first and second internodes at the booting stage.

The control of plant growth and development by DELLA is dependent on GA-regulated growth and developmental responses [44–46]. However, we did not find genes involved in GA metabolism among the DEGs in this study. Instead, the DEGs identified in this study included auxin-, SA- and BR-related genes (Table 1). These results suggested that GA interacts with these hormones [46]. DELLA can directly trigger the expression of auxin- and BR-related genes to affect plant growth [47, 48]. For example, the expressions of *SCP* and *CYP450* (both grouped into BR-related genes) were dramatically down-regulated by the DELLA mutation *Rht-B1b* to potentially cause dwarfism in DPW (Table 1), because the expression of *SCP* positively affects plant growth [49]. Auxin represses the expression of *ARP* genes [50, 51]. In a previous study, overexpression of an *ARP* of *Brassica rapa* caused a reduction in vegetative growth [50]. Auxin also modulates the expression of *ASCO*, which encodes a crucial enzyme that produces oxidative molecules, including H_2_O_2_ [52]. Overexpression of an *ASCO* in cotton enhanced the accumulation of H_2_O_2_ and promoted cell elongation, whereas suppression of an *ASCO* in tobacco and *Arabidopsis* inhibited stem cell growth [53]. Our results show that the DELLA mutation *Rht-B1b* resulted in dramatically up-regulated *ARP* and down-regulated *ASCO* in DPW (Table 1). Auxin-induced growth inhibition is accompanied by decreased levels of reactive oxygen species [54]. Thus, the accumulation of the DELLA mutant protein regulated via auxin-mediated signal transduction may reduce the contents of reactive oxygen species such as H_2_O_2_ [41], thereby limiting cell expansion to cause dwarfism in DPW.

In rice, over-expression of an *early nodulin* gene resulted in improved nitrogen-use efficiency and increased nitrogen assimilation [55]. In C_3_ plants, nitrogen assimilation is positively correlated with phosphoenolpyruvate carboxylase (PEPC) phosphorylation [56, 57], which is catalysed by phosphoenolpyruvate carboxylase kinase (PPCK). The extent of phosphorylation is largely determined by PPCK activity, which is controlled by the level of *PPCK* transcripts [56, 58, 59]. A reduction in PEPC activity leads to serious stunting of growth [60]. Our results showed that the DELLA mutation *Rht-B1b* led to significant down-regulation of *early nodulin* and two *PPCKs* in DPW (Table 1). Thus, decreased nitrogen assimilation and PPCK activity may decrease the activity of PEPC [43, 59] to cause dwarfism in DPW.

The hemicellulose xylan and pectins are two abundant polysaccharides in the plant cell wall [61]. Their modifications, such as methylesterification and acetylation, have been proposed to influence cell wall architecture and function, causing various plant growth phenotypes [61–64]. Our results showed that the DELLA mutation *Rht-B1b* led to significant down-regulation of the expression of several pectin-related genes, including *PEL*, three *SBTs,* and *α-Gal* (Table 1). Decreases in the transcript levels of these genes may lead to the repression of pectin degradation and the accumulation of de-esterified pectin [63], enhanced pectin methylesterase activity to stiffen the cell wall [65], and reduced adherence of pectin to the cell wall [66]. Thus, the DELLA mutation *Rht-B1b* may result in modifications of pectin that limit cell wall loosening and inhibit cell elongation, thereby causing dwarfism in DPW.

Many studies have reported that either excess or inadequate acetylation of xylan disrupts the cell wall structure, thereby causing dwarfism in plants [67, 68]. Our results show that the DELLA mutation *Rht-B1b* up-regulated eight xylan acetylation-related genes, including three *GDSL esterase/lipase* genes, two *ESKIMO* genes, *IRX15-L*, *AXY-L,* and an uncharacterized acetyltransferase gene (Table 1). ESKIMO and AXY-L are xylan acetyltransferases, and IRX-L is involved in synthesis of the xylan backbone [61, 67–70]. A specific interaction between acetyltransferases and xylan backbone biosynthetic enzymes may repress acetylation of adjacent residues [68, 70]. Therefore, even though the transcript levels of *ESKIMO*, *IRX15-L*, *AXY-L* and IRX-L were up-regulated (Table 1), the acetylation of xylan might be decreased. GDSL esterase/lipase is a xylan deacetylation enzyme [64]. The DELLA mutation *Rht-B1b* resulted in up-regulated expression of *GDSL esterase/lipase*, leading to enhance xylan deacetylation. Therefore, the DELLA mutation *Rht-B1b* may reduce acetylation of xylan to limit cell wall loosening and inhibit cell elongation, causing dwarfism in DPW.

**Fig. 7:**
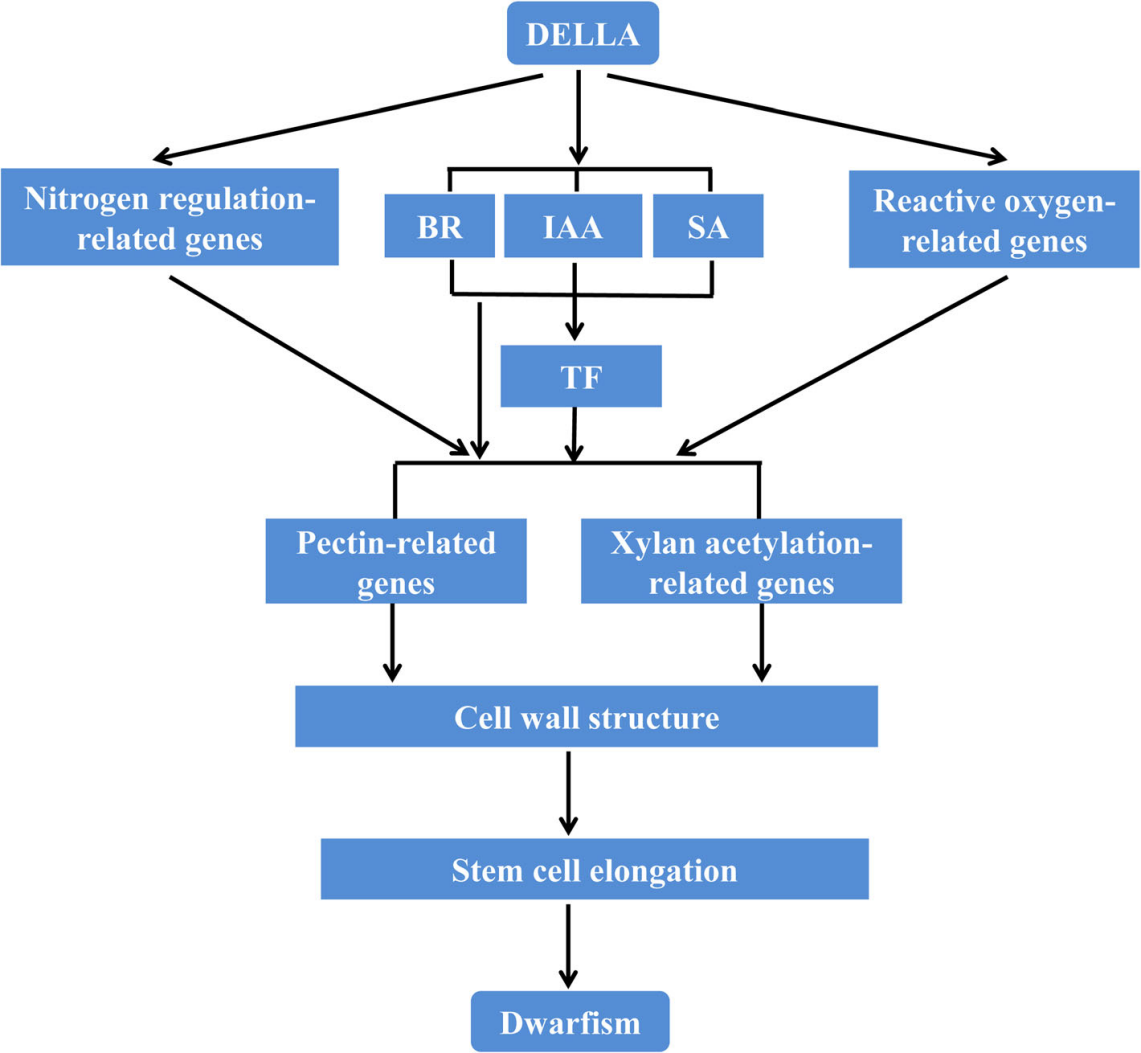
Molecular network model of the DELLA mutation *Rht-dp* in DPW

A model summarizing how the DELLA mutation *Rht-dp* causes dwarfism in DPW is proposed (Fig. 7). Whether the DELLA mutation *Rht-B1b* regulates the pathway of hormones, reactive oxygen species, and nitrogen assimilation, it ultimately affects the cell wall structure to limit cell wall loosening and inhibit cell elongation, thereby causing dwarfism in DPW.

## Conclusion

In summary, our results indicated that the semi-dwarfing gene *Rht-dp* is the“Green Revolution” gene *Rht-B1b*. It regulates pathways related to hormones, reactive oxygen species, and nitrogen assimilation to modify the cell wall structure, and then limits cell wall loosening and inhibits cell elongation, thereby causing dwarfism in DPW.

## Supplementary Information

**Additional file 1: Fig. S1.** The plant height of DPW, TPW, and DPW × TPW F_1._

**Additional file 2: Fig. S2.** Relative expression of *ARP* and *ASCO* in the first and second internodes at the booting stage.

**Additional file 3: Table S1.** The information of 59 tetraploid wheat accessions.

**Additional file 4: Table S2.** The information of SSR primers on 4BS chromosome.

**Additional file 5: Table S3.** Genotype data of RIL populations and the F_2_ population from D_60 × T_58.

**Additional file 6: Table S4.** Gene-specific primers for *Rht-dp* candidate genes in DPW and TPW.

**Additional file 7: Table S5.** The information of Dwarfism-related DEGs induced by DELLA mutant *Rht-dp.*

## Data Availability

All data generated or analyzed during this study were included in this article and the supplementary files.

## Abbreviations

BR: Brassinolide
cM: centimorgan
DEGs: Differentially expressed genes
DPW: Dwarf polish wheat
FPKM: Fragments per kilobase of transcript per million mapped reads
GA: Gibberellin
Jianyangailanmai: Ailanmai
LOD: Logarithm of odds
NIL: Near-isogenic line
qPCR: Quantitative real-time PCR
RIL: Recombinant inbred line
RNA-seq: RNA sequencing
SA: Salicylic acid
TPW: Tall polish wheat
