# Supplementary figures and images for: The semi-dwarfing gene Rht-dp from dwarf polish wheat (Triticum polonicum L.) is the "Green Revolution” gene Rht-B1b
Source: BMC Genomics. 2021 Jan 19;22:63. doi: 10.1186/s12864-021-07367-x (PMC7814455; doi:10.1186/s12864-021-07367-x)

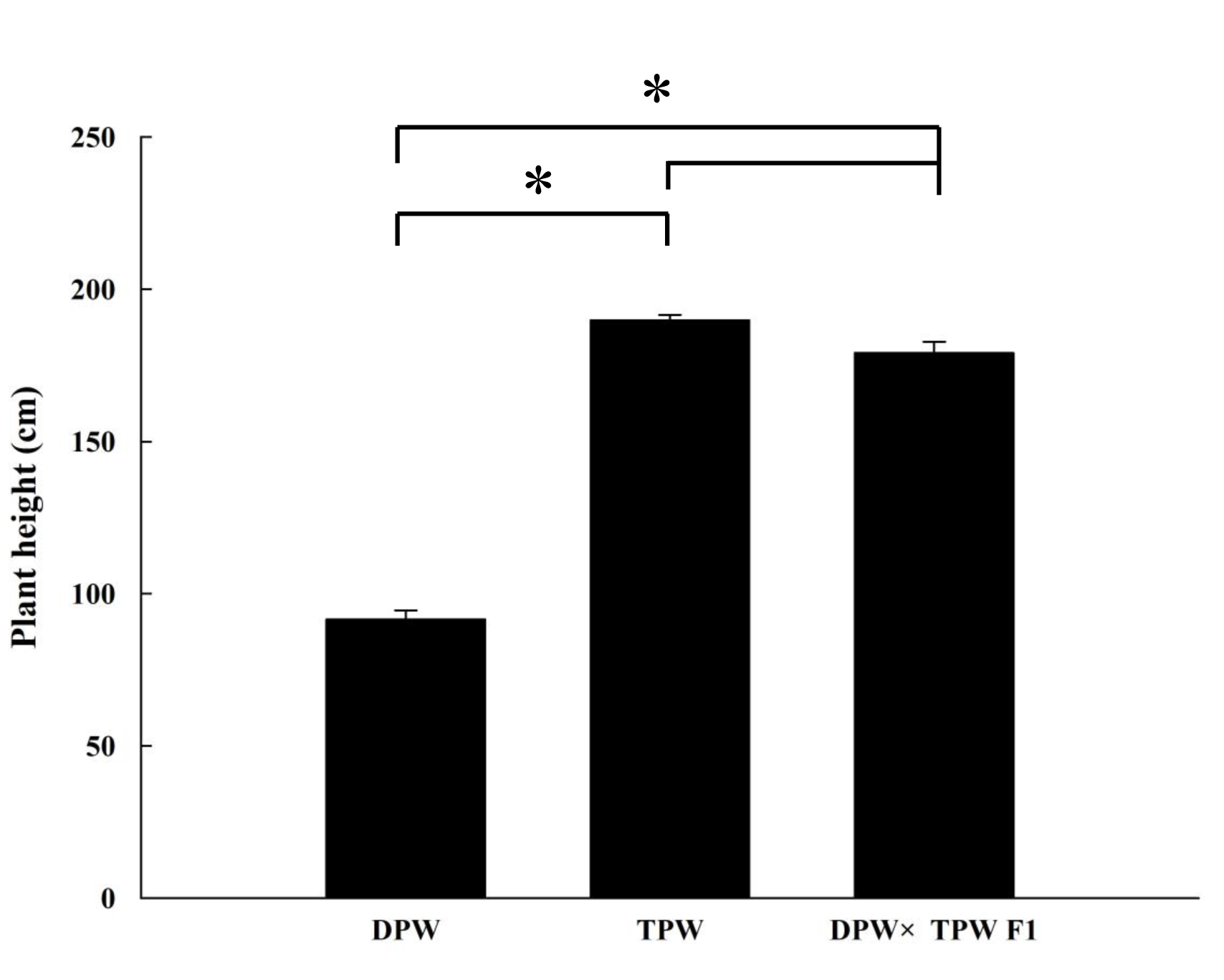

Supplement: Supplementary file 1 — Additional file 1: Fig. S1. The plant height of DPW, TPW, and DPW × TPW F1. [file 12864_2021_7367_MOESM1_ESM.tif]

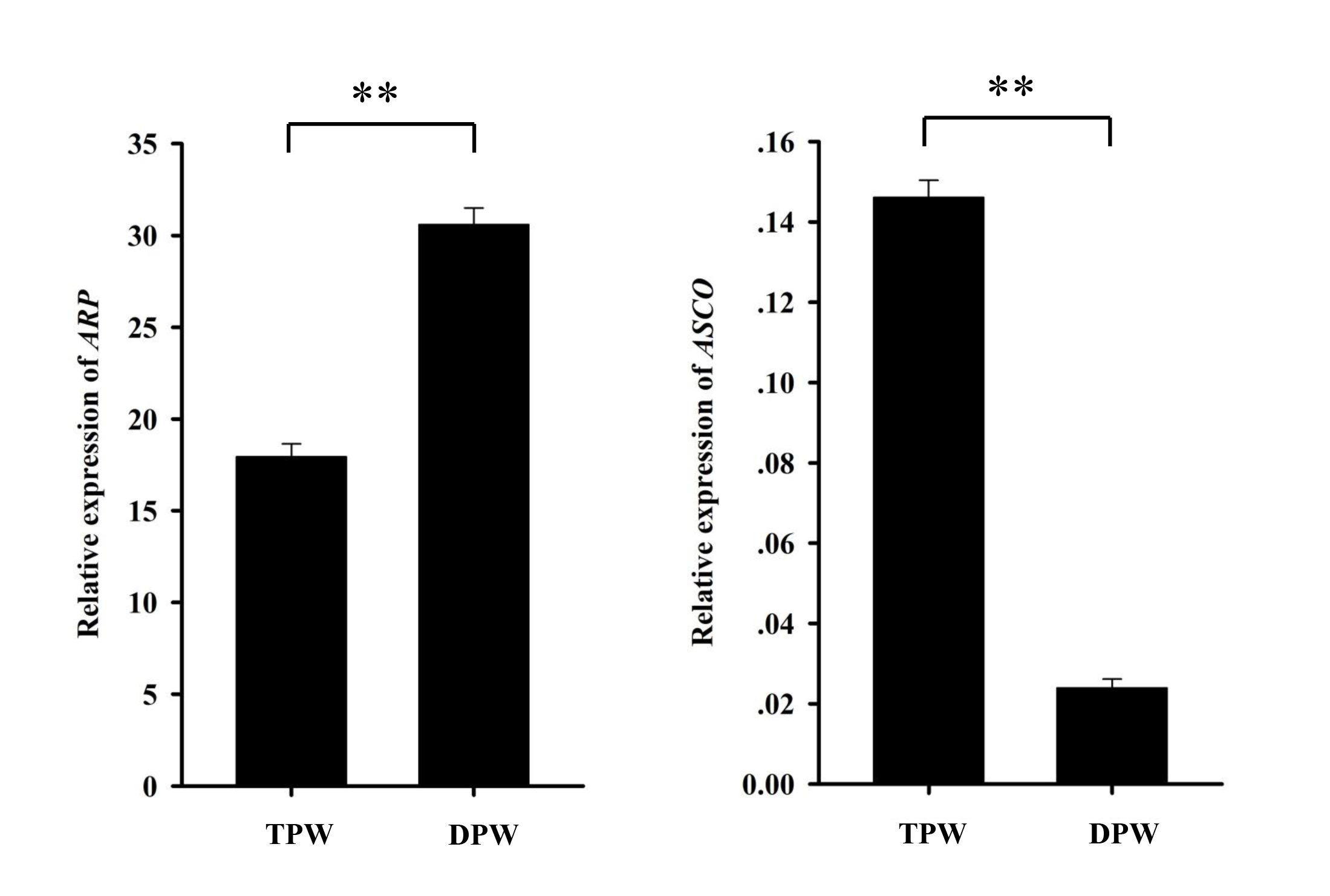

Supplement: Supplementary file 2 — Additional file 2: Fig. S2. Relative expression of ARP and ASCO in the first and second internodes at the booting stage. [file 12864_2021_7367_MOESM2_ESM.tif]
