# Supplementary material for: The semi-dwarfing gene Rht-dp from dwarf polish wheat (Triticum polonicum L.) is the "Green Revolution” gene Rht-B1b
Source: BMC Genomics. 2021 Jan 19;22:63. doi: 10.1186/s12864-021-07367-x (PMC7814455; doi:10.1186/s12864-021-07367-x)
Supplement: Supplementary file 3 — Additional file 3: Table S1. The information of 59 tetraploid wheat accessions. [file 12864_2021_7367_MOESM3_ESM.pdf]

Table S1 The information of 59 tetraploid wheat accessions

Number	Storage code	Cultivar	PH (cm)	Haplotype	GenBank
1	AS2236	*T. turgidum*	144.6	*Rht-B1r*	MN551138
2	AS2238	*T. turgidum*	151.7	*Rht-B1a*	
3	AS2239	*T. turgidum*	110.8	*Rht-B1r*	MN551139
4	AS2240	*T. turgidum*	146.5	*Rht-B1r*	MN551140
5	AS2255	*T. turgidum*	130.7	*Rht-B1r*	MN551141
6	AS2285	*T. turgidum*	147.5	*Rht-B1r*	MN551144
7	AS2291	*T. turgidum*	143.5	*Rht-B1r*	MN551145
8	AS2295	*T. turgidum*	142.5	*Rht-B1r*	MN551146
9	AS2296	*T. turgidum*	166.8	*Rht-B1r*	MN551147
10	AS2298	*T. turgidum*	143.5	*Rht-B1r*	MN551148
11	AS2299	*T. turgidum*	145.1	*Rht-B1r*	MN551149
12	AS2305	*T. turgidum*	161.5	*Rht-B1r*	MN551150
13	AS2308	*T. turgidum*	147.5	*Rht-B1r*	MN551151
14	AS2310	*T. turgidum*	156.8	*Rht-B1r*	MN551152
15	AS2312	*T. turgidum*	160.5	*Rht-B1r*	MN551153
16	AS2313	*T. turgidum*	158.2	*Rht-B1r*	MN551154
17	AS2334	*T. turgidum*	151.6	*Rht-B1q*	MN551155
18	AS2351	*T. turgidum*	147.2	*Rht-B1r*	MN551130
19	AS2378	*T. turgidum*	149.2	*Rht-B1r*	MN551156
20	AS2380	*T. turgidum*	147.1	*Rht-B1r*	MN551157
21	AS2381	*T. turgidum*	142.5	*Rht-B1r*	MN551158
22	AS2382	*T. turgidum*	152.1	*Rht-B1r*	MN551159
23	AS313	*T. turgidum*	110.8	*Rht-B1r*	MN551160
24	ZH2232	*T. turanicum Jakubz*	141.1	*Rht-B1q*	MN551133
25	PI124494	*T. turanicum Jakubz*	137.8	*Rht-B1a*	
26	PI184526	*T. turanicum Jakubz*	137.9	*Rht-B1h*	
27	PI184543	*T. turanicum Jakubz*	129.5	*Rht-B1u*	MN433368
28	PI211691	*T. turanicum Jakubz*	134.8	*Rht-B1h*	
29	PI306665	*T. turanicum Jakubz*	134.8	*Rht-B1h*	
31	PI191781	*T. dicoccum Schrank*	136.5	*Rht-B1t*	MN433367
32	PI221401	*T. dicoccum Schrank*	139.3	*Rht-B1s*	MN551165
33	PI350001	*T. dicoccum Schrank*	146.1	*Rht-B1s*	MN551166
34	PI352358	*T. dicoccum Schrank*	147.9	*Rht-B1a*	
35	PI352367	*T. dicoccum Schrank*	141.5	*Rht-B1h*	
36	PI352369	*T. dicoccum Schrank*	149.5	*Rht-B1h*	
37	PI355465	*T. dicoccum Schrank*	128.5	*Rht-B1h*	
38	PI355507	*T. dicoccum Schrank*	128.5	*Rht-B1q*	MN551131
39	PI377655	*T. dicoccum Schrank*	136.5	*Rht-B1s*	MN551163
40	PI434998	*T. dicoccum Schrank*	141.8	*Rht-B1s*	MN551164
41	PI94614	*T. dicoccum Schrank*	134.5	*Rht-B1r*	MN551162
42	PI94650	*T. dicoccum Schrank*	126.5	*Rht-B1a*	
43	ZH2235	*T. dicoccum Schrank*	103.1	*Rht-B1a*	

44	ZH2237	*T. durum*	87.5	*Rht-B1a*	
45	Langdon	*T. durum*	126.5	*Rht-B1r*	MN551161
46	LD222	*T. durum*	146.5	*Rht-B1q*	MN551132
47	AS2262	*T. durum*	137.2	*Rht-B1r*	MN551142
48	IC12196	*T. polonicum*	91.1	*Rht-B1b*	
49	AS304	*T. polonicum*	91.5	*Rht-B1b*	
50	AS302	*T. polonicum*	189.9	*Rht-B1q*	MN551135
51	CNG8389	*T. polonicum*	183.1	*Rht-B1q*	MN551136
52	CNG8391	*T. polonicum*	175.6	*Rht-B1q*	MN551137
53	CNG12289	*T. polonicum*	178.1	*Rht-B1a*	
54	CNG12291	*T. polonicum*	181.5	*Rht-B1a*	
55	PI294478	*T. Ispahanicum Heslot*	134.1	*Rht-B1h*	
56	PI350048	*T. Ispahanicum Heslot*	132.3	*Rht-B1h*	
57	AS2268	*T. persicum Zhuk*	156.5	*Rht-B1r*	MN551143
58	ZH2331	*T. persicum Zhuk*	142.1	*Rht-B1q*	MN551134
59	ZH2334	*T. dicoccoides Korn*	128.7	*Rht-B1a*	

* PH: Plant height.
