# Supplementary material for: The semi-dwarfing gene Rht-dp from dwarf polish wheat (Triticum polonicum L.) is the "Green Revolution” gene Rht-B1b
Source: BMC Genomics. 2021 Jan 19;22:63. doi: 10.1186/s12864-021-07367-x (PMC7814455; doi:10.1186/s12864-021-07367-x)
Supplement: Supplementary file 4 — Additional file 4: Table S2. The information of SSR primers on 4BS chromosome. [file 12864_2021_7367_MOESM4_ESM.pdf]

Table S2 The information of SSR primers on 4BS chromosome

Number	Primer name	Forward primer	Reverse primer
1	*Xgp 8A5D*	GCAGCAACAACTCTTCGACA	TGCCCTTCTGTGGCTAGATT
2	*Xgpw0103*	CATGATGAGGAGGGAGTAGTTC	TCAAAGGGACAGACTCAGTTC
3	*Xgpw0110*	TCAATTCCAGCACGGTTACA	CGAGGGCTTGAAATGAACAAC
4	*Xgpw0457*	CGGATATTTAAGAAACGGTGAAGG	GAGGTGTTGGTGAGGATGAC
5	*Xgpw0703.1*	CTTGATGGCACGAGGATGA	CAACACTGGTAGCAGGATATAGAG
6	*Xgpw0722*	GCCAGAGAGGACCAAAGATG	GCGAGAGGGCAGTCATATTC
7	*Xgpw08F3*	GTCTTCTTCGGCAGACAAGG	CAACCCCATCGGTAAACAAG
8	*Xgpw0916*	CGGTGTGCGTGTGATATAGA	GAGATGGTTGTTGTGTTACAGATG
9	*Xgpw0CD3*	TCTTTTGCGGGGAAAGAATA	ACATGGGTCAGGCAGCTTAC
10	*Xgpw11ED*	TATGAATGGATCCACAGCGA	CAACGCAGATACACACACCC
11	*Xgpw1221*	AAACGAACATCTACATCATGCA	TTTAACACGCACAACACACA
12	*Xgpw122700.1*	AGCCTCTCTGATGTGCAAGG	CCCAACACCATCGTCCTAAT
13	*Xgpw122900.1*	AAGTTATCGGGTCTGCGATG	CTTTTGTCCCGGTAGCTTTG
14	*Xgpw123000.1*	AGGACGGTGAGGAAGAGGAC	ATGGGGTCCCCTAAATCAAA
15	*Xgpw123100.1*	TAGCAAGCATCACCTCATGG	AACAGGCTCTTTTCCCTGGT

16	*Xgpw123800.1*	GATGAAGGCTTCCACCAAAA	CATGGGATCTCTTTGTCGGT
17	*Xgpw123900.1*	AGTGCATCATGTCGAAGCAG	GATTCGGCTAGTAGCAGCG
18	*Xgpw124500.1*	GGCCTCACCTTCTTCTTCCT	GGAGACAGATGCGGATGAAT
19	*Xgpw124600.1*	TTGTAGGCGGGAAAAGAGAA	TTGATTGATGTGTGTGCGTG
20	*Xgpw124800.1*	TTTTACCATTTGCCTCAC	GACCAACACTTAGACAAG
21	*Xgpw125500.1*	CTCACCACCACCTATATTTC	GGACCAACACTTAGACAA
22	*Xgpw125700.1*	CGAATCCAATATTGTGGAGGA	CGGTGGCTTTAGTCATGTTG
23	*Xgpw126000.1*	TGCCTCCAGCATAGTCACTG	AGGGCAGGAAAAAGTGGAAT
24	*Xgpw126300.1*	GCTTGCCTTATATTTTGGGGA	GAGTTGGTGATGCTCCCATT
25	*Xgpw126500.1*	TATGCTGTTGGGAGATGCAG	GCATCGGCAATACAACTTCC
26	*Xgpw126500.2*	TAATCCCTTCCCACATGCTC	GGGAGACAGGAGGTTCAACA
27	*Xgpw127200.1*	CACAAAGGAGCAGCAAATCA	AACATCTGTCTTGCGTGCAG
28	*Xgpw127400.1*	GACTGCAGTATCCTGTGGCA	TCCTGAGGAAGATACGGGTG
29	*Xgpw128400.1*	GCCAACTCTTGCTTTTGAGC	ACGTTGTGCTCTTCGCTTTT
30	*Xgpw128500.1*	TGACACCGAGGTTTGCATTA	CAGCAGATGCTTCAACCAAA
31	*Xgpw128600.1*	GCCCCTTCCTTGAAGTTGTT	TTCACGCTCCTAGCACATTG
32	*Xgpw129200.1*	GAGAAGGAGGCGAGGAGTTC	CGATATTCATGCTGCAATGG

33	*Xgpw130400.1*	GGCAATCAATCGGAAGAAGA	CCAAGGATGTGGTTCTGGTT
34	*Xgpw131100.1*	TTCATGCTCATCGTGGTTTC	GAGGGTACACTCCACCATGC
35	*Xgpw131200.1*	GAGCATTTGCCTGCTCTTTC	GAGTACCACAAGCACCCGAT
36	*Xgpw131400.1*	GTTAGTTGGGTCAGGCGAAG	CTAGCAGGGATGGGATTTGA
37	*Xgpw131500.1*	TTCCGGCTTTAATTTGCTTG	AGCAACCCCTTTGCCTTAAT
38	*Xgpw132500.1*	TGGCTCATGGACGAGTATCA	CAGCAACCTGTTCTTGTGGA
39	*Xgpw132700.1*	GGAGGAGGTAGAGGAGGAGG	TTTATCGCTCCCATCTTTGG
40	*Xgpw133100.1*	GCCTCCTCCAACACCATCTA	TCCGTCCATAAAAACTTGCC
41	*Xgpw133200.1*	AGGAGCGGAGGGAGTAATGT	GAGCAGAGCACATCAGTTCG
42	*Xgpw133500.1*	GCTCACTCCTCTTCCGTGTC	AAAGAAAACGACCGATTCCA
43	*Xgpw133600.1*	GCCATCTACGCTGACAGTGA	TTCTTGTTCCAACCAGGAGG
44	*Xgpw134000.1*	TACATGCAACTGATCCAGCA	GGTCCTCGTCGCTGAAGTAG
45	*Xgpw134200.1*	AGTAAGCACCAATGCCATCC	TGCCAGCAGTACAAAGTTCG
46	*Xgpw135000.1*	GATGATTTCGCATTCCCATT	GCCTTGTGCTCTTTGGACTC
47	*Xgpw135700.1*	CTAGGAAGATTGCACCGAGC	TCTGTAAATCTCCGATGCCC
48	*Xgpw135800.1*	GTCTCGCTCGACAGGCTC	GCCTGGTGGTAGAAATGGAA
49	*Xgpw135900.1*	AGGTAGCTCCATTCCTGCAA	TGTTTGTGATGTGGCTCGAT

50	*Xgpw136000.1*	ACGGTGGGGAGCTAACCTAT	GGACTTGTTGCGGAAACATT
51	*Xgpw145500.1*	GCCAGATTGATTCCATCCTC	GTCACCCCAAACTCCTCGTA
52	*Xgpw145600.1*	GAGTACGAGGAGGGCTACGA	CGGTTCAGCTAGACCGCTAC
53	*Xgpw148100.1*	TCCCAGATGCCAATGAATTT	GGAGGCCAAAAGGCTCTAGT
54	*Xgpw1483.1*	AATTTCAAAGTCATGTTCTCGGTTT	CAATGCCTGTGTGTTGCTAC
55	*Xgpw148600.1*	GCAGCAGAGGATGAAGCAG	AGGTAAGGAGCCCACATGC
56	*Xgpw1566*	TGCCGAATAACCACTGTTGA	CCGTTCATTTGTAGTGGGGT
57	*Xgpw1AFB*	CCGTGCGCAGATATAGAACA	AGCATCTGTGCTGACAGTGG
58	*Xgpw1D85*	TCTACCACTGCAATGCAACC	AACTCTCTCTCCTCTCCGGC
59	*Xgpw2161*	TAAGATCCAACTACAATAGCAAAGC	CCATGATGAGGAGGGAGTAG
60	*Xgpw2800.1*	AGAGCGCCATAGCGATACAC	TTGGCTGATTATTTTTCCCA
61	*Xgpw2830.1*	AAGGATTTGGGACGGAGTTT	GAGAGGAACCAAGAATGTAGGA
62	*Xgpw2871.1*	CTTGTCGCCGTCCAACCT	GGATCGTCGGCTGCTAGT
63	*Xgpw2970*	GCAGGAGGAGTGGAGAAG	AGAGAGAAAGAAAGAGGCAAAGA
64	*Xgpw2977.1*	GGGCTGAGGGTATGTACTAG	ACATCAAGATCGTGCCATAGA
65	*Xgpw2994.1*	CAGTGTCTGACCATTATTAGCA	CTGTCTAGTCTGTGTACTCTCC
66	*Xgpw29F6*	ATCAGCATGTGCAAAACCAA	CATTTTCAAGGCGGCATATT

67	*Xgpw2F9F*	CTGGGGACGCTTTTGTTTTA	GATCAGTTCCGCTTCTCCAC
68	*Xgpw3006.1*	ATCAATGGGCATCAGGTAGG	CCTTTGCGACGACTTTCTG
69	*Xgpw3008.1*	TTCCGCTTTGGACTGTCTAAA	TCGTGCATTGGTCGTCTC
70	*Xgpw3035.1*	CCTCTAACCACAAGCACTCT	GGTCTAGGACTAGGAGGAGATAA
71	*Xgpw3080.1*	GCATATTAGAGCCAACTTAGGTAA	GAAGGAGAAGGAGAAGAACAATG
72	*Xgpw3090.1*	ACCTACCTACAATGTTTGACTATCG	CCTTATTCTCTGCTACACATATCCA
73	*Xgpw3111.1*	TGTGTTGGAGCGATTCAGAT	TTTCGCCTCCCAACCTTT
74	*Xgpw3118.1*	CCATAGAGAGAACACGAGAGAG	TATCCATGACGAGGAGGAAGTA
75	*Xgpw3120.1*	GCGGAAGGATCTCATCAAC	CGTCACTCATCGGAGGAG
76	*Xgpw3121.1*	CGGAGGTGTTGGTGAAGAT	GCAGAATCAGGGAACGTAGAA
77	*Xgpw3128.1*	GCATACTACCAGACCAGTGTT	ATCTATCTGACGGGCTTGTG
78	*Xgpw3129.1*	GGAGAAGGAGTGGATCTATCA	CTCACTTTGAACAAACCCTAGA
79	*Xgpw3131.1*	CGTTGGAGAAGTGGTTGGT	GTATGTGAAGATGAGCAAAGTGAAG
80	*Xgpw3132.1*	CTGACTGACTGACTGACTGAC	GATCCCTTTCGCCATCTCAG
81	*Xgpw3134.1*	CACTGGTGTCCATCTTCATCAT	GGGAGAAACATCATAAGATCCAACT
82	*Xgpw3140.1*	CGAGAGTTTTGTGTGAGAGA	ACCACTAAAATTAGTCTCTCTC
83	*Xgpw3149.1*	GGCAAAGATAACCGGATTACAAG	CTGCTAATTCGCCAAGTCCA

84	*Xgpw3151.1*	CCGCTAACCGAAAGAAAGAA	GTAGGTATACCCCGTTGTGA
85	*Xgpw3152.1*	TGGCATAGTATGGTACCGTAC	TTGGGACTAAAGGCTTTGTTG
86	*Xgpw3154.1*	CCACGATGAGGAGGGAGTAG	GCCCATGATATAGATCACGAGAG
87	*Xgpw3156.1*	GGTGTTTGAAATCATCATTCTTA	AAGGCAGCTTGAAGAATTAG
88	*Xgpw3157.1*	ACGAGTATGATGGCGACGAG	GCTCATTTACTCCACTCAACCTC
89	*Xgpw3160.1*	TCCGTTGTTGCTCTTCATTG	CGGGTTGAGTTTAGCATAGAG
90	*Xgpw3163.1*	GTTGGTAGGTTCGCCTCA	GGGAATGCGTAGGGAAAGA
91	*Xgpw3173.1*	TGGGCTAGATGGAGAAATGTTA	CCTAGAACCAACTCAACTGTC
92	*Xgpw3298.1*	AACCTTGTTGCCCTTCTGAT	CGAATAGAACTCCAAGAACATCAAG
93	*Xgpw3325.1*	GCCATCACCTCGTCTTCTAT	CGGTCGGCTTCTCCTATC
94	*Xgpw3390*	AACATTGCACCTTCTCACCTT	CTTCACGCTTGCTTGCTAGT
95	*Xgpw3427.1*	AGTCAGTCAACAACACCAAGA	CCACATTCATTCCGTTCATAGAC
96	*Xgpw3447.1*	CCCTGTTCACCTGCTCTC	ACTTCATCCGTAGTAACCATTTGT
97	*Xgpw3472.1*	GCAAGTTCAGGACGAGGAA	TCTCCAATGGCAAGTAGATGTC
98	*Xgpw34A2*	GGGGCCTGCCTAATTTATTC	AGAAGGGGAACAGAGGTCGT
99	*Xgpw3536.1*	TCATCGGAGAGTATTAGAACAGTG	TCAGTAGCAAGTGCAGTACATAA
100	*Xgpw3552*	GACTCCTCCTCTGAATCCATT	AACCTGGTCACTACATCTCTG

101	*Xgpw3852*	ATTTCCTCCCACATTAGTTCATCT	CAGGGTGGAAGAAACTTACAGA
102	*Xgpw3862.1*	TTGGCTGGCCTCGCTAAA	GGCGATGCCTCAAGATGG
103	*Xgpw3868*	TTGTACAGTAGTAGTATCGATGGAT	TTTATTTGAGGAGTGGGTTGTG
104	*Xgpw3957.1*	ATCCGCCGCAGAAACATC	GCTCGCTCCTACACTAACC
105	*Xgpw3BCD*	GATCTGGACCTCTGCCTGAC	TAAACCAAGTTGCACGTCCC
106	*Xgpw3C28*	TCCTCCTTTCCCTCCCTAAA	CAGTGGCGAATCTAGCATGA
107	*Xgpw3DC4*	CCTCTTTGTCGGAGAAGTGC	TTTCCAAAACCCGATAGCAG
108	*Xgpw4047.1*	CGATTCAGTGCCAAGTCA	GTCGTCGATGTCCCTCTC
109	*Xgpw4049*	ATGGTAATGGGATTGCTGAGT	CTGCCGACCTAGAACCTATC
110	*Xgpw4064.1*	AACCAGCAAACCAAGACAAC	CACCATGCCGACTCTCTC
111	*Xgpw40AA*	GGCACACTGACAGACAGCAT	ATATTTCTCCTCTGCGGCAA
112	*Xgpw40AA*	GATCTGGATGAGGATTCGGA	GATCTGGATGAGGATTCGGA
113	*Xgpw4100.1*	ATGGTTACAACTACAGATG	ATATGGCAAAGAGAAAGC
114	*Xgpw4117*	GCACTGTCCTCTCCAAAAGC	AGTTTGCGACGATAAACGCT
115	*Xgpw4140*	CGAAAATAAGACTCAAATGGGC	AGTGGTTTCTATCCAGCACACA
116	*Xgpw4233.1*	CGTAGCTGTTTATGTCCACCTT	ATGATTGGTCTGGCTGATGAG
117	*Xgpw43B8*	TCGTCGTCTGTCCAGTGAAG	CGGTCGCACCACACTACTC

118	*Xgpw4507*	CCTCTGATGTGGGTGGAAGT	GTCGGCCTATAAGACCCCAT
119	*Xgpw4564*	CCAGGTAAGTGTCCCAGGTG	GTTGAGGTCTGTTGGAAATAATTCT
120	*Xgpw4800.1*	GAGGTTCGCATTTCACAG	GGTTCTGACCATCACTTG
121	*Xgpw4900.1*	CTTCCAGGAATATGTTAGGT	CTCGAATTCCACCATGTG
122	*Xgpw4C7D*	CACCGTCACATCACATCACA	CACTTCAGGATGTTCCCCAT
123	*Xgpw5000.1*	CTTCACAGTCTGTCTCTC	ACCCAGTTACAACAATCA
124	*Xgpw5032.1*	GAACAAGTATCATCATCACACAACA	CTGCTGCTGCTCTTCTCT
125	*Xgpw5200.1*	AACAGTACAAGGGAAGAAC	GATCCGCATCTGAGAGAG
126	*Xgpw5300.1*	CAGGTAACTCGCTATATTGAT	TGGCAAAGTAGATCCTTC
127	*Xgpw5400.1*	GCCTCTGTGCCACTGAAC	CCACCTGTCATGCTCTGTTAG
128	*Xgpw5500.1*	TCATAGGTTCTTGGGTAG	ATGACTGGTAAGCTAGTT
129	*Xgpw5500.2*	GGATTTAGGAAGGACTGT	CTTGGTCATCTCCTCTAC
130	*Xgpw5600.1*	GAGGAAGGAGAAGGAGAAG	AGGAAACTAAGCATATTTGAGATAA
131	*Xgpw5700.1*	GCTATAAGTGAATGTTGTAGAGG	CTTCGCATTTGGTTTGTCT
132	*Xgpw5800.1*	AGATCCAACTATAATAGCA	CTATGTGTTTAACTCTCTC
133	*Xgpw5900.1*	TACTTAACTTGTCAGTCTTA	TCATCCTCGTCTTCATCT
134	*Xgpw6000.1*	AAATCAACGAGAAACTTAACTTA	TGGTTTAGTTTAGTTTAATTTAGGT

135	*Xgpw6003*	CTCTGCGTTAGGACTCTATACAA	TCCCCTCTAATAGAAAACATGATGA
136	*Xgpw6102*	GGAGAAGTAACCAAGTGGATGAG	GCCCATGTACCCTTTCATTCT
137	*Xgpw6124*	TCAGACCCACCGCATAAATC	GGGTAGGGAGATGGTTTGTC
138	*Xgpw6241*	GGCAACAGCATAGAGGGTAC	AAGAGGAATAGGGAGAGGAGTTT
139	*Xgpw6332*	CAGGCGAGGACTGACTTTAG	TGACGAATAGTACAAGGAAGAACAA
140	*Xgpw673D*	TGCCTCGTTCCCTTATTTTG	TCACCATTGTTTCCACAGGA
141	*Xgpw674C*	ACACCATCCTATGCTCTCGG	CACGATAGTCAACATCCCCC
142	*Xgpw6884*	TTCCTTTGCTACTTCTGCTAACT	CTCCACCGTCTACTGTTCATC
143	*Xgpw6895*	CATTGGTCCGTCTTATGGTAGAT	GTCAAGAACCACCTGGAAGT
144	*Xgpw6973*	CAAATGGACTGGCACATACAAAT	GGTGAACAATATGGAGGTGAGAA
145	*Xgpw7189*	GGCATGGATGAGGTCATAGG	GCTAGTTTTGCTTGCCTTGG
146	*Xgpw719B*	TGGTATTGTTGCTGTCTGCTTT	GCAATGGCTACAATGTCCAG
147	*Xgpw7322*	AGACACCAACCAAGGAGACG	TCATAAGGTTGGGGAGCAAG
148	*Xgpw735F*	CTGACCTCTCGTCACACAGC	ATCGTTCTGGGTTGTTGGAG
149	*Xgpw7363*	GCTCGGTTGGTACAAGATTG	CACACTCAGTTCGTCTCTCT
150	*Xgpw7581*	TGACCACGTTTTTGTTGAGC	TGCTCTTTGGGGTGTTTAGG
151	*Xgpw7762*	CGAGCACACAACTCCAATTC	GAGTAGGAGGTCTTGCCTTC

152	*Xgpw7847*	ATGCTTTGATTTCTTCCCTGAG	GACGGTATAGACGACGATGG
153	*Xgpw8200.1*	CTGTTCTTTCATTGGTCGCA	CGCACAGATAAATGCAATGG
154	*Xgpw83E7*	ATTCTACACCCCAGGAACGA	TGTCTCTTTCCTTGTACCCCA
155	*Xgpw8412*	TCTATTGCCTTACTCATGGATT	CGTAGTGCATCATGTCGAAG
156	*Xgpw8716*	CCTACCATTGTAACCGACTCTAT	CGTCCTGCTTGATTGATATTGAT
157	*Xgpw8748*	CCCAATCCGATGAAACTAATGAAG	CGAGCCTAAACCATAATTCCCTTA
158	*Xgpw8773*	TCCTCAAGCAAAAGCCGATA	GATGTTGTGATGAATCGTTCCTT
159	*Xgpw8A5D*	ACAGAATCTGCAGGTCCCAC	CACCACACCACCAATCTCTG
160	*Xgpw8AA9*	AAGTGGATCTGGCCTCCTTT	GTGGTACACGGTTTCGGTTT
161	*Xgpw8B93*	CCGAAGTTCGTCATCAATCA	AGCAGCTCAAAAAGGACGAG
162	*Xgpw8BA4*	GTCGATTGCTGGGTGAGTTC	CAGAGCAGAATGCAGAGACG
163	*Xgpw8F56*	TAAATGCTGCCACAAAGTGC	TTTGACATGGCTTCATTCCA
164	*Xgpw9337*	CTCACATGCACCCTTTGCTA	GCATTTTGGAGCATCGATTT
165	*Xgpw9371*	TACTCACGGTTGCTTTGCTA	CCTGGGACCTCCTAACTACT
166	*Xgpw9422*	TAAAAACCAGACCAAACCCG	GTGGAAGGCGACCTCAGAT
167	*Xgpw9508*	TCGTTGTTGTTCAGAGGAGAG	GGAAAGATTAGCAGCCCAGT
168	*Xgpw9586*	CTATGGCGGAGCAACCTTAT	CCGGTTAATACAATTCTAGCATGAA

169	*Xgpw9952*	GCTACAACAACAACGCTTACAA	GTCATAGGTCCAAGTGGTGTC
170	*Xgpw9D35*	TCAAAATGATGTGATGCGGT	TGTCAACAAAAAGATACGCTAAGA
171	*Xgpw9F54*	GGTCTTAGGTGCCAAGCAAA	TTTGGAACTGCCCTAAGCTG
172	*XgpwA396*	AGGGTCCTGGCAGATATCAA	CAATTCGTGATCATTGGTGC
173	*XgpwA567*	TGCTGGAACTTAGCAGCAGA	ATGTCCTTTTCTCGTGGTGG
174	*XgpwA82D*	CATGGGATCTCTTTGTCGGT	GATGAAGGCTTCCACCAAAA
175	*XgpwAB31*	CCGGTCTATCGTCATCAGGT	CAGAAGAGGGTGAGGAGCAG
176	*XgpwAC29*	TGCGTACAGAGATCAAGCTGTT	CTCCTGAACCCAGCAAGAAG
177	*XgpwB07F*	GAGGCAAAATGTAGCCTTCG	TCTCCTTCTTCCTCGAGCTG
178	*XgpwB4A3*	TCCAGTGGGCAGAGCTACTT	GTATTCTGGACCCTGGGTGA
179	*XgpwB902*	GGTTTTCAAGCCTGATGGAG	ATGTTCATCCCAGGAAGTGG
180	*XgpwBA65*	CGCTACTCCATTTCTCCAGC	TATTCGCGTGCAATTTGGTA
181	*XgpwBB50*	CCTGTTTGACATTTTGCTGC	GAGATGTCCAACTCGTGGGT
182	*XgpwBCA7*	TTGAGCTGCCAAATTGTTGA	ACTGCTGATGACGCTGGAAT
183	*XgpwC20E*	ATCGAATGGGAATTTTGACG	CGCTCGAAGGGAGATAACAG
184	*XgpwCB41*	GCCTGAATTCCAGTGGCTAA	ATTTCGGTTCATCTTGCGTC
185	*XgpwCDA2*	AAACAGCAGTTGACCCTTGG	GGATTGTGAGAACCCACCAC

186	*XgpwCEC2*	ACCAACGACGAAAACTCTGG	GTCGATCATGCCTCTTGGAT
187	*XgpwD080*	TAACTCGCAAGTGGCACAAC	CGCCAGGGCAGTTAAGATAG
188	*XgpwD9C9*	TTCCTATCAAACTGGGGCAG	TCGCTGACAAATTCTTGTGG
189	*XgpwE1EA*	GAAAGCAGTTCCATTCGAGC	TCAGTGCTCCACCAATAAACC
190	*XgpwE58A*	CTCTTCCTGCGAAACTACGG	TTACAATTTGAGCTGTGGCCT
191	*XgpwE6E4*	ATGTAGGGAAAGGCTGCGTA	TCCACCCATTAGCTTTGTCC
192	*XgpwE81D*	TCTACCACTGCAATGCAACC	AACTCTCTCTCCTCTCCGGC
193	*XgpwF574*	CCCTACTCAGCACCTAACCG	GGTGAACGGACAGAAGCACT
194	*XgpwF66A*	CTTCAACCAGCTGCAATCAA	GAGTGGGAGTTGGATGGAGA
195	*XgpwF7D1*	AAGAAATCAGGGGTCCCATC	CGTATGGGTAGAATGTCGGG
196	*XgpwFCFB*	ACGAAGAGCAGAAGCTGGAC	TGCCATTCCTTTCTCTCGTT
197	*XgpwFD88*	TTAATTACGCGACCAAACGG	CACGGAAGTGCCACTCACT
198	*XgpwFF39*	CGGATTCATCAAGGTCATCC	CCGTCGATGCAGTCCTCTAT
199	*XgpwFFA9*	TGGTGCAGTAGATGGAGCAG	CAAATCCAATCGAAACCATTC
