# Supplementary material for: The semi-dwarfing gene Rht-dp from dwarf polish wheat (Triticum polonicum L.) is the "Green Revolution” gene Rht-B1b
Source: BMC Genomics. 2021 Jan 19;22:63. doi: 10.1186/s12864-021-07367-x (PMC7814455; doi:10.1186/s12864-021-07367-x)
Supplement: Supplementary file 6 — Additional file 6: Table S4. Gene-specific primers for Rht-dp candidate genes in DPW and TPW. [file 12864_2021_7367_MOESM6_ESM.pdf]

**Table S4 Gene-specific primers for *Rht-dp* candidate genes in DPW and TPW**

| Number | Primer Name | Forward Primer | Reverse Primer |
|---|---|---|---|
| 1 | *DELLA protein* | CGATGCCGTCTACAACTACT | CAACTCCTAGATCGGGAAACTT |
| 2 | *DELLA protein-QDZ1* | AGTCACTCAAGCGGATATGC | CACCATCATCTTGTCCTCGG |
| 3 | *DELLA protein-QDZ1* | CGGTTCCTCTTCTTGACTCG | GCATATCCGCTTGAGTGACT |
| 4 | *TB1-1* | GGTGTTTGAAATCATCATTCTTA | AAGGCAGCTTGAAGAATTAG |
| 5 | *TB1-2* | GGAGAGTATCACATCCAATATTCTA | CTCGATCAATGCTTGTTAGTATATG |
| 6 | *TB1-QDZ* | GCCTAAACCCATCCATAATC | GGGAATCATAGAAAGGAAACAT |
| 7 | *Zinc Finger Protein-1* | TTCGCCTTCAACTAACATTAGAGA | GGGGCAGACCGTAACAAA |
| 8 | *Zinc Finger Protein-2* | TCGGCGGTGTCAACTTTG | GCCAATGTGCTATGTAAGCAATG |
| 9 | *Zinc Finger Protein-3* | GCTGTCTACAATACACACTTGA | CCACTAATATAGAACAACATGAAGG |
| 10 | *Zinc Finger Protein-4* | CTCAGAATCAGTGTCGTTGC | ACTCCCTCCTCCCTATCC |
| 11 | *Zinc Finger Protein* | TCTGCTTCACGCTCCTCC | CCACCAACACCACCAGAATTG |
| 12 | *Zinc Finger Protein-QDZ* | CGAGGAGGAGCGTGAAGCAGAA | CCTGTCGGCGGTGACTGTTT |
| 13 | *42800-NA* | GCAGGCCCTTAACAATGCTA | GTGACTAGTACTGGTACAACAGATG |
| 14 | *42800-QDZ* | GCTACTGTCAACCCGTAACT | CCTTGTCTGGTTCGCTAGT |
| 15 | *EamA-like* | CGTCATCTACGGTAATACCTACTA | GCCCAATCTAAACCATTCCTC |
| 16 | *EamA-like-1* | TTCCCCAAACACTTTCAGAC | ACAGCAGCATAATGGATATCG |
| 17 | *EamA-like-2* | GTAGCTATCAACAGTGATACTCTG | CAGGCATTGGATTATCTCTAGTC |
| 18 | *EamA-like-QDZ* | TGCTCTTTAAGTTTCGGTTCGTAAT | GGTGTGAATGTGGCGATGAG |

*Gene sequences were downloaded from wheat genome Refseq v1.0 (IWGSC 2018)
